# Preparation and Characterization of Liposomes Containing Essential Oil of *Eucalyptus camaldulensis* Leaf

**Published:** 2012-08-25

**Authors:** Eskandar Moghimipour, Nasrin Aghel, Ali Zarei Mahmoudabadi, Zahra Ramezani, Somayeh Handali

**Affiliations:** 1 Nanotechnology Research Center, Ahvaz Jundishapur University of Medical Sciences, Ahvaz, IR Iran; 2 Medicinal Plant Research Center, Ahvaz Jundishapur University of Medical Sciences, Ahvaz, IR Iran; 3 Infectious and Tropical Diseases Research Center, and Department of Medical Mycology, Ahvaz Jundishapur University of Medical Sciences, Ahvaz, IR Iran

**Keywords:** *Eucalyptus camaldulensis*, Essential Oil, Liposomes, Antifungal Activity

## Abstract

**Background:**

The increased incidence of fungal resistance has necessitated the need to search for new antifungal agents.

**Objective:**

The main objectives of the present study were to investigate the effectiveness of the essential oil of *Eucalyptus camaldulensis* on dermatophytes growth and to formulate and characterize a liposomal gel loaded with the essential oil.

**Materials and Methods:**

The essential oil extracted from the leaves of E. camaldulensis was analyzed by GC-MS. The antifungal activity of this essential oil was determined against Microsporum canis, M. gypseum, Trichophyton rubrum and T. verrucosum, using the well diffusion method. Liposomes were prepared by the freeze-thaw method and evaluation of size distribution was performed using a particle size analyzer. The liposomal gel was prepared using ‘hydroxethyl cellulose (HEC) as the gelling agent. The rheologic characteristics were determined by a Brookfield viscometer.

**Results:**

The results showed that the minimum inhibitory volume of the essential oil was 0.125 ml and 95 ± 0.57% of the essential oil was successfully entrapped in the liposomes. The main constituents of the essential oil detected by GC-MS were; phenol, 1, 8 cineole, limonene, alcohol, pinene and terpinen. Results of particle size determination showed a wide range from 40.5 to 298 nm for the different formulations. No significant thixotropy was observed in the rheogram of the formulated liposomal gel.

**Conclusion:**

Liposomal gel formulation of the essential oil may lead to improved antifungal activity.

## 1. Background

In recent years, the incidence of infections caused by dermatophytes has increased considerably. Essential oils are natural substances extracted from plants with proven antifungal effects. *Eucalyptus camaldulensis* (Myrtaceae) is a plant indigenous to eastern Australia and Tasmania which is also cultivated in southern Europe and many other regions of the world (1). Essential oil of Eucalyptus is a colorless or pale yellow liquid (1) that contains; cineol, terpenes, sesquiterpenes, aromatic aldehydes, phenols, pinene and limonene (1). Eucalyptus oil is frequently used for the treatment of coughs and nasopharyngeal infections; it can also be used as an antiseptic and expectorant. It has been shown that an ethanolic leaf extract of this plant has a marked fungicidal effect against dermatophytes (2). Due to a lack of stability of most of the essential oils, new methods have been developed to improve their stability, among these is the encapsulation of the essential oils in liposomes (3). Liposomes are vesicles composed of concentric phospholipid bilayers (4). Due to their capability to deliver slow drug release, cutaneous targeting and extended transdermal delivery of drugs, liposomes have been reported to be promising drug carriers for antimicrobial therapy (3).

## 2. Objectives

The main objectives of the present study were to investigate the effectiveness and encapsulation of the essential oil E. camaldulensis, and to formulate and characterize a liposomal gel loaded with the essential oil. Some parameters such as; vesicle size, entrapment efficacy and stability of the formulation were also investigated.

## 3. Materials and Methods

### 3.1. Plant Materials

The leaf of E. camaldulensis were collected from Ahvaz (Iran), and indentified in the Department of Pharmacognosy, Faculty of Pharmacy, Ahvaz Jundishapur University of Medical Sciences.

### 3.2. Preparation of Essential Oil

500 g of fresh leaf samples of the plant were cut into small pieces. Then, the essential oil was extracted using a distillator for 3 h and stored in a refrigerator for future use.

### 3.3. GC- MS Analysis of Essential Oil

To identify the main constituents of the essential oil, its GC-MS analysis was performed on GC 7890A equipped with MS 5975C detector and HP-5ms capillary column (30 m × 0.25 m, 0.25 µm) (Agilent Company, USA). The initial column temperature was set at 60º C, then increased from 60º C to 190º C with 5º C̸ min, from 190º C to 270º C for 30º C min, and finally kept at 270º C for approximately 5 min, so that the total analysis time was about 34 min. The total ion chromatogram of Eucalyptus essential oil was analyzed by the GC-MS head space. To acquire this chromatogram, a GC vial contain Eucalyptus essential oil was put in the head space cavity and after shaking it for approximately 3 minutes at 150° C, 25 ml of volatile compounds from the above sample was injected into the GC running the above mentioned program.

### 3.4. Antifungal Activity

Freeze-dried sealed glass ampoules of microorganisms were obtained from the Persian Type Culture Collection (PTCC), Iranian Research Organization for Science and Technology, Tehran, Iran. The microorganisms were; Microsporum canis PTCC no. 5069, M. gypseum PTCC no. 5070, Trichophyton rubrum PTCC no. 5143 and T. verrucosum PTCC no. 5056. These microorganisms were activated on Sabouraud dextrose broth (SDB) and then cultured on Sabouraud dextrose agar (SDA) for 21 days to obtain adequate growth. For determination of the antifungal activity, a well diffusion method was utilized. Each plate was inoculated with 50 µL of the fungal suspension. Various serial dilutions of the essential oil were prepared and 50 µL of each serial dilution transferred to plates and incubated at room temperature for 3 weeks. The Minimum Inhibitory Concentration (MIC) was determined to be the lowest concentration of the essential oil that did not show any viable growth after 3 weeks of incubation (5).

### 3.5. Preparation of Liposomes

Liposomes were prepared using a freeze-thaw method. Soya lecithin and cholesterol (1:1) were dissolved in chloroform and methanol (100 mg/ml). The solvent was removed by rotary evaporation under vacuum. Then phosphate buffer saline (pH 7.4) and essential oil (0.25%) were added and mixed in a vortex for 5 min. The solution was frozen in ice-ethanol or acetone (5-10 min) and left to thaw at room temperature. The freeze-thaw cycle was repeated three times and then the samples were centrifuged at 10 000х g for 30 min (6-9).

### 3.6. Determination of Encapsulation Efficiency

Cineol, the major component of E. camaldulensis oil, was chosen as an index for determination of encapsulation efficacy. It was assayed according to the method previously described by British Pharmacopeia (determination of cineol). After the samples were centrifuged, the supernatant was removed and the liposomes were treated with Triton X-100 to disrupt them. Then 3 g of the substance was added to 2.10 g of melted o-cresol, this was allowed to cool by stirring continuously. Then the mixture was re-melted and allowed to cool again. The highest temperature at which the mixture froze was then recorded (10).

### 3.7. Measurement of Liposome Size

The diameter of the liposomes before and after homogenization (for 15 min) was determined using a particle sizer, Qudix, ScatterO Scope I system (Korea).

### 3.8. Scanning Electron Microscopy (SEM)

The specimens were dried under light and coated with a silver layer. Then they were examined photographically by a scanning electron microscope (Leo 1455 VP, Germany, magnification 10 000x).

### 3.9. Preparation of Liposomal Gel

Hydroxyethylcellulose (HEC) 5 g, was slowly added to a PBS buffer solution (pH 7.4), and stirred constantly with a paddle stirrer. After the addition of the full amount of solid material, the gels were allowed to swell under moderate stirring for at least 4 h to achieve maximum volume and transparency. Finally 0.4 g of the liposomes was added (11).

### 3.10. Determination of Rheological Properties

The rheologic behavior of the sample was determined using a Brookfield viscometer (model DV-I with No. 34 spindle). The viscosity of the samples was determined at 0.3, 0.6, 1.5, 3, 6, 12 and 30 rpm for 1 min at room temperature. The results were plotted as a rheogram and their rheologic behavior was determined by fitting these on the corresponding Newtonian and non-Newtonian equations.

### 3.11. Stability Testing

The stability of the vesicle dispersions was monitored after 1 and 3 months storage in a refrigerator. At certain time intervals, the liposomes were evaluated for their encapsulation efficiency and size distribution.

### 3.12. Statistical Analysis of Data

The data were reported as mean ± SD and frequently as percentages. For all of the analyses, the statistical significance was assessed at a level of 0.05 and a t-test was used to compare the averages.

## 4. Results

**Figure 1:**
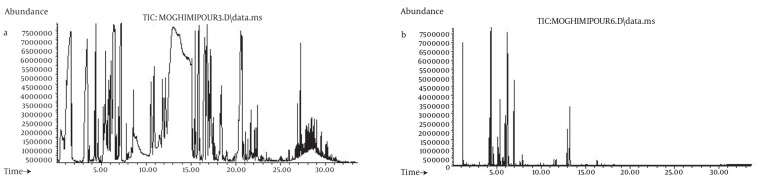
a) Total Ion Chromatogram of GC-MS Analysis of Essential Oil (1 μl injection in split mode). b) Head Space GC-MS Analysis of Essential Oil as Described in the Text.

**Figure 2:**
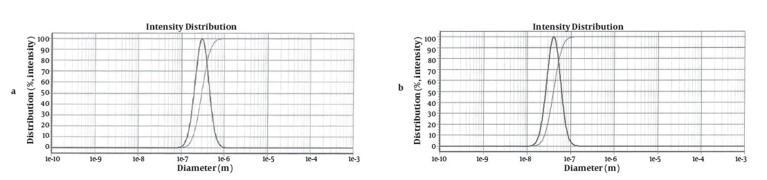
a) Particle Size Distribution of Non-homogenized Liposomes Containing Essential Oil of *Eucalyptus camaldulensis*. b) Particle Size Distribution of Liposomes Containing Essential Oil of *Eucalyptus camaldulensis* After Homogenization

The yield of the essential oil of E. camaldulensis was 2% v/w. GC-MS analysis of the essential oil showed that it contained; phenol, 1.8 cineol limonene, alcohol, pinene and terpinene (Figure 1a, b). The minimum inhibitory concentrations of the essential oil for all of the micro-organisms were 0.125 ml. The results showed that 95 ± 0.57% of the essential oil was successfully encapsulated in the liposomes (n = 3). The results of the size monitoring showed the effectiveness of freezing time on the size of the particles. There was a significant increase in the particle size of the liposomes. There was also a significant rise in the polydispersity of the particles (Table 1). Homogenization time was also significantly affected by the liposomal particle size, as indicated in Figure 2a, b.

**Figure 3:**
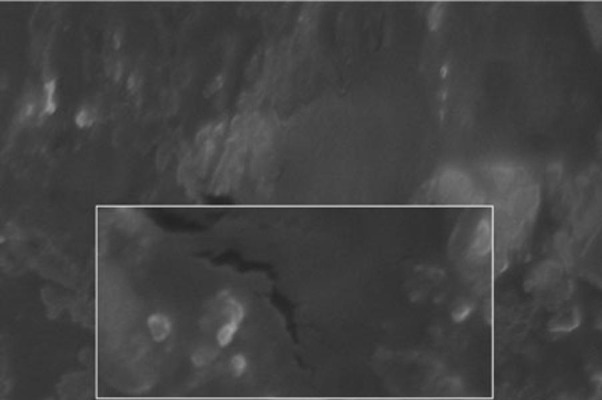
SEM Image of Freeze-thaw Liposomes Containing Essential Oil of *Eucalyptus camaldulensis* (Magnification 10000x)

**Figure 4:**
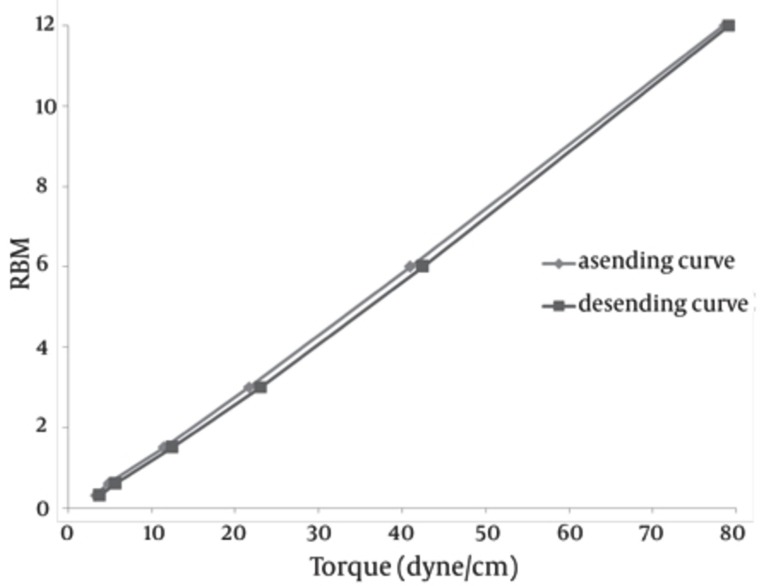
Rheogram of Liposomes Containing Essential Oil of E. camaldulensis at Room Temperature

The SEM image of the liposomes containing essential oil of E. camaldulensis, confirmed the formation of spherical particles and also monodispersity of particles, as shown in Figure 3. Rheological inspection of the formulations showed mild pseudoplastic to Newtonian behavior with no significant thixotropy (Figure 4). The results of the stability study showed good incorporation efficiency of the essential oil after one and three months of storage (94 ± 0.57%) and showed no significant size change (P > 0.05). Results of particle size and encapsulation before and after storage are shown in Table 2.

**Table 1:** Effect of Freezing Time on the Particle Size of Liposome (n = 3)

Formulation	Freezing time, min	Particle size, nm	Polydispersity index (PDI)
F^1^	4	157.66 ± 0.57	2.5 ± 0.0
F^2^	8	158.66 ± 0.57	2.58 ± 0.14
F^3^	10	286.33 ± 2.88	2.66 ± 0.14
F^4^	45	36430 ± 1.25	1.41 ± 0.94
F^5^	> 60	290000 ± 6.65	2.05 ± 0.42

**Table 2:** Results of Particle Size and Encapsulation of Essential Oil of *Eucalyptus camaldulensis* Before and After Storage

Characterization	Before storage	After storage
Particle size (nm)	157.66 ± 0.57	156.33± 1.15 a
Encapsulation (percent)	95 ± 0.57	94 ± 0.57 a

^a^P > 0.05

## 5. Discussion

The development of fungal resistance to presently available antifungal agents has necessitated the need to search for new antifungal agents. Essential oils possess a wide spectrum of biological activity in several fields, from food chemistry to pharmaceutics. However, most essential oils are biologically instable, poorly soluble in water and they are distributed ineffectively to the target sites. New methods have been developed in order to improve their stability, among these is the use of liposome bilayers to encapsulate the oil (3).

The compounds are revealed in the total ion chromatogram (TIC) shown in Figure 1a. When a split injection of the Eucalyptus essential oil is performed on the specified column, a broad peak appears around retention times 11 to 14 min. In order to simplify the spectra and distinguish the compounds more precisely, a head space injection was also performed and its TIC was recorded (Figure 1b is a less crowded chromatogram). This allowed better estimation of the compounds present in the essential oils, which then became the mechanism used in the library search, by comparing the two TIC (Figure 1a and Figure 1b) RT of 4.37, 5.397, 5.984, 6.970, 7.90, 12.957, 13.256 that are related to α-pinene, gamma-pinene, α-terpinene gamma-terpinene linalool, and phenols respectively. The results obtained by GC-MS analysis of the essential oil showed that it contained phenol, 1, 8 cineol, limonene, alcohol, pinene and terpinene. It had previously been reported that the major components of the essential oil of E. camaldulensis were; ethanol (25.36%), eucalyptol (13.73%), β-caryophyllene (11.55%) and carvacrol (9.05%) (12). In another study, the composition of this plant consisted of 1, 8 cineol (64%), α- pinene (9.6%), myrcenol (7.4%) and γ-terpinene (7%) (13).

The results of the present study confirmed the antifungal properties of the essential oil from E. camaldulensis on dermatophytes. Several previous studies have investigated the antibacterial and antifungal properties of E. camaldulensis. It has been shown that the essential oil of the E. camaldulensis leaf and E. globulus leaf effectively inhibits the growth of S. aureus and E. coli. (14). In another report, methanolic extract of E. camaldulensis had been formulated as an antidermatophytic cream preparation (15). Another study showed the antitermitic activity of oils of E. camaldulensis leaf against Coptotermes formosanus, it was demonstrated that the termiticidal mechanism was due to inhibition of acetylcholinesterase activity (16).

In the preparation of the liposomes, the length of the freezing period affected particle size. With a time of more than 60 min, particle size of the vesicles increased, while in shorter time frames, particle size of the liposomes decreased significantly (Table 1). So it is suggested that the time of freezing is an important parameter in creating the size of the liposome. With more than 60 min of freezing time, the particle size distribution shows polydispersity and aggregation, while there is a decrease in polydispersity due to shorter freezing times. According to our data, after homogenization the particle size of the vesicles decreased to 40.5-298 nm. The results of one study showed that poloxamers P338 and P407 inhibited the particle growth observed during the freeze-thaw cycle for egg PC MLVs dispersed in 1.0 M NaCl, probably through steric prevention of aggregation and fusion (6). The results of another study demonstrated that by increasing time sonication, particle size decreases from 969 nm to 677 nm, but the particle had polydispersity (17). Liposomes have been reported to be promising drug carriers in antimicrobial therapy, with targeting and low transdermal delivery of drugs and many other investigations carried out in different fields. The quantity of essential oil encapsulation in liposomes was 95 ± 0.57%. Ortan et al. have reported that the molar ratios of lecithin and cholesterol influence the drug entrapment capacity of the liposome. It has been previously shown that by using phospholipid PC in the formulation, incorporation of Anethum graveolens essential oil in the liposome was 98% (3). According to the results of another study, only 4.16% of 1.07 mg pure carvacrol was successfully encapsulated into the liposomes (18). In another report, the percentage of lidocaine HCL encapsulation into liposome gel was over 72%. According to our stability data of vesicle dispersions, there was good incorporation efficiency. Also, no significant changes were observed in the particle size of liposomes. Liposomal incorporation of carvacrol and thymol isolated from the essential oil of Origanum dictamnus and in vitro antimicrobial activity has been studied previously, the results showed that antimicrobial activity dramatically increased after the encapsulation in liposomes (18). SEM image analysis of liposomes containing essential oil of E. camaldulensis showed the spherical structure and monodispersity of the particles. Topical application of liposome vesicles has many advantages over conventional dosage forms. It has been suggested that formulated liposomes be applied to the skin as a gel. It has previously been shown that the liposomal gel of lidocaine HCL may perform therapeutically with better effects than conventional formulations (19).

This study concluded that the presence of E.camaldulensis in liposomes may effectively enhance its stability and the entrapped oil remains stable for an extended period of time. Liposomal gel formulation of essential oils may also lead to improved and better antifungal activity.
